# Gut metabolites: key factors in the cross-talk between the gut microbiota and tumor immunotherapy

**DOI:** 10.3389/fimmu.2026.1882542

**Published:** 2026-07-22

**Authors:** Lizhu Zeng, Yuxin Ren, Hui Huang, Qiurong Wang, Huimin Li, Jieyu Song, Feng He, Jun Li

**Affiliations:** 1 School of Clinical Medicine, Chengdu Medical College, Chengdu, Sichuan, China; 2 Department of Gastroenterology, First Affiliated Hospital of Chengdu Medical College, Chengdu, Sichuan, China; 3 Laboratory of Infectious and Liver Diseases, Institution of Infectious Diseases, West China Hospital of Sichuan University, Chengdu, China

**Keywords:** gut microbiota, metabolic reprogramming, microbial metabolites, tumor immunity, tumor microenvironment (TME)

## Abstract

This review synthesizes recent research findings and proposes an integrated “microbiota–metabolite–immune–oncology” framework, highlighting how gut-derived metabolites regulate the dynamics of tumor immunity and informing the development of next-generation immunotherapies. Key metabolites—including short-chain fatty acids (SCFAs), bile acids (BAs), trimethylamine N-oxide (TMAO), indole-3-propionic acid (IPA), and urolithin A—exert bidirectional effects on antitumor immunity through multiple mechanisms. These include histone acetylation-driven epigenetic reprogramming, aryl hydrocarbon receptor (AhR)- and farnesoid X receptor (FXR)-mediated metabolic reprogramming, and direct regulation of immune effectors such as CD8^+^ T cells and myeloid-derived suppressor cells. Emerging evidence highlights specific roles of these metabolites within the tumor microenvironment (TME): microbial dysbiosis can amplify immunosuppressive circuits, whereas targeted enrichment of certain metabolites may enhance the efficacy of immune checkpoint blockade. Integrative multi-omics analyses have revealed the vascular remodeling effect of TMAO and the spatiotemporal heterogeneity of BAs, thereby connecting the gut-liver-tumor axis and achieving overall immune regulation. By mapping a precision-oriented metabolic roadmap, this review identifies underexplored therapeutic avenues—such as metabolite-targeted interventions and engineered probiotics—that, when combined with immune checkpoint inhibitors, may enable personalized, microbiome−based strategies with the potential to improve outcomes in cancer immunotherapy.

## 1 Introduction

Gut microbiota-derived metabolites have emerged as important signaling molecules that transcend local host-microbe communication to systemically shape the tumor microenvironment (TME) (1).Molecules such as bile acids (BAs), trimethylamine N-oxide (TMAO), and short-chain fatty acids (SCFAs) can influence the trajectory of antitumor immunity and modulate the efficacy of immune checkpoint blockade (ICB) therapies. Beyond their role in oncogenesis, these metabolites markedly modulate the immune landscape across multiple axes. For instance, BAs can bidirectionally regulate regulatory T cell (Treg)/T helper 17 (Th17) cell differentiation via the farnesoid X receptor (FXR) and Takeda G protein-coupled receptor 5 (TGR5), and metabolic imbalances in this axis often promote a tolerogenic niche (2, 3). TMAO can potentiate CD8^+^ T cell effector function through type I interferon signaling, though its activity is concentration-dependent (4). In parallel, SCFAs can epigenetically remodel tumor-infiltrating lymphocytes through inhibition of histone deacetylases (5).

Beyond metabolites, structural microbial components known as microbe-associated molecular patterns (MAMPs) also exert potent immunomodulatory effects within the TME. A prominent example is lipopolysaccharide (LPS), a major endotoxin derived from the outer membrane of Gram-negative bacteria. LPS activates the Toll-like receptor 4 (TLR4) pathway to induce CXCL1 production, driving the accumulation of CXCR2^+^ polymorphonuclear myeloid-derived suppressor cells (PMN-MDSCs) (6) and reinforcing immune evasion through upregulation of S100A11 (7).

Additional metabolites further expand this regulatory network. The tryptophan derivative indole-3-propionic acid (IPA) sustains the stem-like properties of CD8^+^ T cells through the aryl hydrocarbon receptor (AhR) pathway (8). Conversely, phenylacetylglutamine (PAGln) acts as an immunosuppressive metabolite by directly impairing the secretion of effector cytokines, including IFN-γ and TNF-α, by CD8^+^ T cells. This suppression accelerates T-cell exhaustion and promotes resistance to ICB therapy (9).

Despite these mechanistic insights, leveraging the metabolism-microbiota axis for clinical immunotherapy faces several translational hurdles. The spatiotemporal heterogeneity of the gut metabolome means that specific metabolites exert highly context-dependent effects, which can vary considerably between gastrointestinal malignancies and distant extra-intestinal tumors, such as prostate cancer or melanoma. Furthermore, therapeutic outcomes are influenced by the dynamic interplay between host genetics and baseline microbial profiles, while physiological discrepancies between preclinical animal models and human patients continue to limit the direct applicability of intervention strategies.

**Figure 1:**
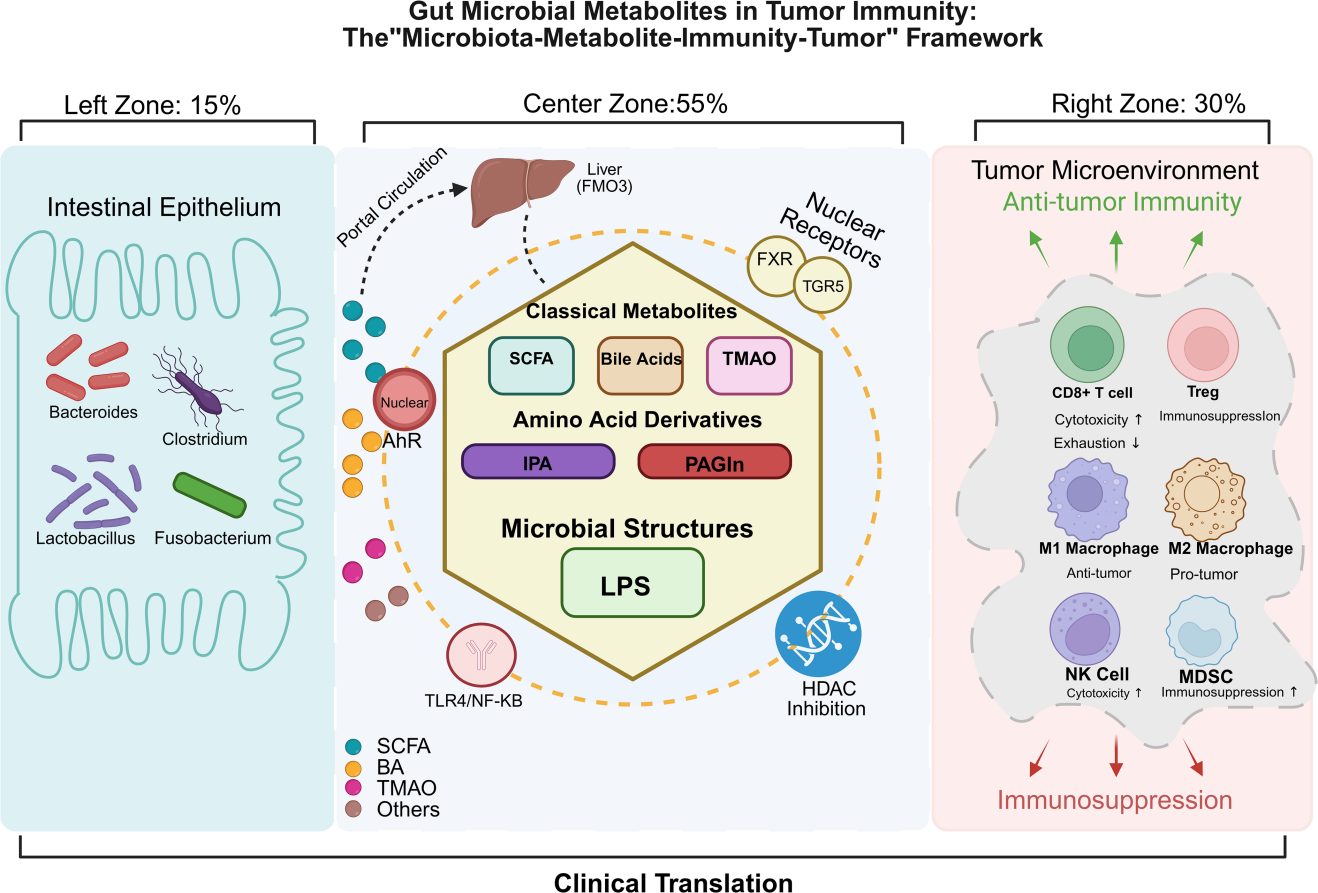
The Integrated Framework of Gut Microbiota-Metabolite-Immunity-Tumor axis. Intestinal commensal and pathogenic bacteria produce diverse bioactive metabolites and structural components, which enter systemic circulation via portal vein after liver metabolism. These microbial products interact with multiple host receptors and epigenetic regulators to bidirectionally modulate antitumor immunity and immunosuppressive landscape within tumor microenvironment, laying a theoretical basis for clinical translational research. (AhR, aryl hydrocarbon receptor; BA, bile acids; CD8^+^ T cell, cluster of differentiation 8 positive T lymphocyte; FMO3, flavin-containing monooxygenase 3; FXR, farnesoid X receptor; HDAC, histone deacetylase; IPA, indole-3-propionic acid; LPS, lipopolysaccharide; MDSC, myeloid-derived suppressor cell; M1/M2 macrophage, type 1/type 2 tumor-associated macrophage; NF-κB, nuclear factor kappa-B; NK cell, natural killer cell; PAGln, phenylacetylglutamine; SCFA, short-chain fatty acids; TGR5, Takeda G protein-coupled receptor 5; TMAO, trimethylamine N-oxide; TLR4, toll-like receptor 4; Treg, regulatory T cell.).

This article systematically reviews the mechanisms by which gut-derived metabolites, together with MAMPs such as LPS, drive immune evasion and modulate ICB responses. By focusing on the TME, we aim to highlight the potential of these microbial molecules as predictive biomarkers and precision therapeutic targets for next-generation immunotherapies (**Figure 1**).

## 2 Definition and classification of intestinal metabolites

Growing evidence indicates that the gut microbiota regulates tumor immunity and immunotherapy responses through several biologically active molecules.

Gut microbiota-derived metabolites help coordinate a complex host-microbe interaction network via receptor-mediated signaling, epigenetic regulation, and metabolic remodeling. In the context of oncology, these molecules serve as critical mediators shaping the TME (9).

Currently, the most extensively studied immunomodulatory metabolites include SCFAs, BAs, and TMAO. SCFAs—mainly acetate, propionate, and butyrate—are produced by microbial fermentation of dietary fiber and function as key immunomodulatory molecules (9, 10).To date, the regulatory roles of these canonical metabolites in tumor immunity have been gradually uncovered along with advancing research. These findings have contributed to a growing consensus that gut microbiota-derived metabolites may function as key intermediaries linking the gut microbiota to tumor regulatory networks.

BAs represent an important class of metabolic intermediates. The gut microbiota, particularly species with bile salt hydrolase activity such as *Clostridium scindens*, transform primary BAs into secondary BAs via 7α-dehydroxylation (2). *Dysbiosis* characterized by a reduction in *Lachnospiraceae* can disrupt this secondary BA pool, leading to aberrant FXR signaling that frequently compromises antitumor immunity (3).

TMAO, a bioactive compound generated through combined microbial and hepatic metabolism, has been associated with gastrointestinal chronic inflammation and malignant progression in observational studies (11).

Microbial catabolism of dietary amino acids generates signaling molecules that influence immune effector cells. The tryptophan derivative indole-3-acetic acid (IAA) activates the AhR, promoting interleukin-22 secretion and enhancing the expression of antimicrobial peptides such as REG3γ (12). Histamine, produced by *Escherichia coli* and *Lactobacillus* reuteri via histidine decarboxylase, can modulate the local immune microenvironment (12).

Similarly, the phenylalanine-derived metabolite PAGln is predominantly synthesized by commensal species such as *Enterococcus faecalis* (13).

Rather than acting as mere metabolic bystanders, these amino acid derivatives can influence the functional states of immune populations within the TME. Other microbial products, including vitamins (synthesized by *Bifidobacterium, Lactobacillus*, and *Bacteroides species*), gas signaling molecules like hydrogen sulfide (produced by *Desulfovibrio*), and lipid metabolites such as conjugated linoleic acid (generated by *Butyrivibrio fibrisolvens*), collectively maintain intestinal mucosal barrier integrity and basal immune homeostasis (12). While traditionally recognized for their roles in basic physiological processes like energy metabolism and neurotransmission (e.g., GABA and 5-HT), their cumulative impact on barrier integrity and local inflammation underscores their broader relevance in preventing microenvironmental shifts conducive to tumor evasion (9).

Beyond metabolites, intestinal bacteria can also influence host immune responses through MAMPs, thereby contributing to the multidimensional regulation of antitumor immunity. MAMPs are conserved microbial structures recognized by host immune receptors to initiate immune signaling. LPS, a structural component of the outer membrane of Gram-negative bacteria, consists of lipid A, a core oligosaccharide, and O-antigen. It is a prototypical MAMP that can regulate tumor immunity through independent signaling pathways and systemically modulate immune cell polarization (14). The classification, core molecular targets, and key tumor immunomodulatory effects of these major microbial metabolites and MAMPs are systematically summarized in **Table 1**, with complete information on microbial/dietary origins and detailed bidirectional mechanisms provided in **Supplementary Table 2**. This review takes these classic metabolites as the starting point; in subsequent chapters, we further explore other newly discovered functional metabolites, as well as the independent immunomodulatory roles of structural MAMPs typified by LPS.

**Table 1:** Classification of microbial metabolites and their immunomodulatory effects in tumor microenvironment.

Metabolite class	Key examples	Main targets/receptors	Tumor immunomodulatory effects
SCFAs	Butyrate, Propionate, Acetate	GPR41/43, HDAC	antitumor: HDAC inhibition → CD8^+^ T/NK activation. Pro-tumor: STING-Type I IFN suppression in DCs → radioresistance
Secondary Bile Acids	DCA, LCA, UDCA, isoDCA	FXR, TGR5	antitumor: UDCA → reduced oxidative stress, apoptosis. Pro-tumor: DCA/LCA → M2 TAM polarization; isoDCA → Treg expansion.
TMAO	TMAO	PERK, NLRP3, Type I IFN	CRC: M2 polarization, VEGFA-driven angiogenesis. Pancreatic/TNBC: ICD, “cold→hot” conversion, enhanced ICB response
Tryptophan Catabolites	IPA, Kynurenine	AhR, PXR	antitumor: IPA → H3K27ac at Tcf7 → CD8^+^ T stemness. Pro-tumor: Kynurenine → AhR activation, Trp depletion, TLS impairment
Phe/Tyr Metabolites	PAGln, 4-HPA	JAK2/STAT3 (4-HPA)	PAGln: CD8^+^ T IFN-γ/TNF-α suppression, exhaustion. 4-HPA: CXCL3-mediated PMN-MDSC recruitment → ICB resistance
Polyamines	Spermidine, Putrescine	TDG	TDG-mediated DNA demethylation → immunosuppressive TAM polarization
Urolithin A	Urolithin A	ERK1/2-ULK1	Mitophagy induction → reduced T-cell exhaustion, enhanced CD8^+^ T/CAR-T persistence
One-Carbon Cofactors	Folate, Vitamin B12	DNA/Histone Methyltransferases	-Methyl donor → nucleotide synthesis, T cell & macrophage differentiation, redox balance; dual effects (context-dependent).
LPS	Hexa-acylated lipid A, MPLA	CD14/TLR4/MD2	Hexa−acyl (pathogen): NF−κB → MDSC, chemoresistance. MPLA (commensal): weak TLR4 → Treg, mucosal tolerance

SCFAs, short-chain fatty acids; HDAC, histone deacetylase; STING, stimulator of interferon genes; IFN, interferon; DCs, dendritic cells; DCA, deoxycholic acid; LCA, lithocholic acid; UDCA, ursodeoxycholic acid; isoDCA, iso-deoxycholic acid; TAM, tumor-associated macrophage; Treg, regulatory T cell; FXR, farnesoid X receptor; TGR5, Takeda G protein-coupled receptor 5; TMAO, trimethylamine N-oxide; PERK, protein kinase R-like ER kinase; NLRP3, NLR family pyrin domain containing 3; CRC, colorectal cancer; TNBC, triple-negative breast cancer; VEGFA, vascular endothelial growth factor A; ICD, immunogenic cell death; ICB, immune checkpoint blockade; IPA, indole-3-propionic acid; AhR, aryl hydrocarbon receptor; PXR, pregnane X receptor; H3K27ac, histone H3 lysine 27 acetylation; Trp, tryptophan; TLS, tertiary lymphoid structure; PAGln, phenylacetylglutamine; 4-HPA, 4-hydroxyphenylacetic acid; JAK2, Janus kinase 2; STAT3, signal transducer and activator of transcription 3; CXCL3, C-X-C motif chemokine ligand 3; PMN-MDSC, polymorphonuclear myeloid-derived suppressor cell; TDG, thymine DNA glycosylase; CAR-T, chimeric antigen receptor T cell; GSH, glutathione; ROS, reactive oxygen species; LPS, lipopolysaccharide; MPLA, monophosphoryl lipid A; TLR4, Toll-like receptor 4; MD2, myeloid differentiation factor 2; NF-κB, nuclear factor kappa-light-chain-enhancer of activated B cells; S100A11, S100 calcium-binding protein A11.

^a^ For complete information including primary microbial/dietary sources, see **Supplementary Table 2**.

^b^ A cross-tabulation of metabolite effects by tumor type, anatomical compartment, and receptor expression context is provided in **Supplementary Table 1**.

## 3 Main metabolic products in the tumor microenvironment

### 3.1 BAs

#### 3.1.1 Metabolism and immunomodulatory functions of BAs

Primary bile acids (PBAs) are synthesized in the liver and subsequently converted into secondary bile acids (SBAs) by the gut microbiota through specific biochemical reactions, including deconjugation and 7α-dehydroxylation. While traditionally recognized for their physiological roles in lipid absorption, BAs are now recognized as immunomodulators within the TME (9, 15–17). By activating specific nuclear and G protein-coupled receptors—predominantly the FXR and TGR5—BAs can shape the polarization and functional states of infiltrating immune cells. In gastrointestinal malignancies, local accumulation of certain BAs has been associated with immune evasion and the establishment of an immunosuppressive niche that may facilitate tumor progression (18, 19). Of note, the effects of different bile acid species on tumor immunity are not uniform. Deoxycholic acid (DCA) and lithocholic acid (LCA) can promote an immunosuppressive TME through activation of FXR and TGR5 signaling, whereas ursodeoxycholic acid (UDCA) has shown antitumor properties in multiple tumor models (20, 21). Mechanistically, UDCA has been reported to induce direct cytotoxicity, alleviate local oxidative stress, and attenuate pro-inflammatory signaling cascades that might otherwise favor immune tolerance. By remodeling the metabolic and inflammatory microenvironment, UDCA sensitizes prostate tumor cells to death receptor-mediated apoptosis and precludes the establishment of an immunosuppressive TME. This apparent paradox does not reflect a limitation of research, but rather highlights the “context-dependent” nature of BA effects (20).

Specifically, the immunomodulatory functions of BAs are governed by their species (hydrophobic vs. hydrophilic), local concentration, tumor type (gastrointestinal vs. extra-intestinal), and the expression profile of BA receptors (FXR/TGR5) within the TME (18, 19). For instance, excessive BA accumulation in hepatocellular carcinoma drives M2 polarization of tumor-associated macrophages (22), whereas low-dose UDCA in prostate cancer induces apoptosis by mitigating oxidative stress (20). Collectively, these observations argue against a simplistic classification of BAs as either pro-tumor or antitumor factors. Future investigations should integrate spatial metabolomics with single-cell sequencing to delineate the spatiotemporal relationship between BA concentration gradients and immune cell functional states, and to explore patient stratification strategies based on BA profiles (20).

#### 3.1.2 Regulation of BAs by the gut microbiota

The gut microbiota remodels the BA pool via a variety of enzymes. Bile salt hydrolase (BSH) catalyzes the deconjugation of host-derived BAs to generate free BAs (23, 24). Specific commensal consortia further process these free BAs. For instance, species harboring the *bai* (bile acid-inducible) gene cluster mediate the 7α-dehydroxylation of cholic acid (CA) to DCA, and chenodeoxycholic acid (CDCA) to lithocholic acid (LCA) (23). Concurrently, members of the genus *Bacteroides* utilize hydroxysteroid dehydrogenases (HSDHs) to generate unique secondary BA epimers, such as UDCA (23).

The dynamic composition of the microbiome markedly influences the systemic BA pool. Shifts in microbial diversity—whether driven by aging or specific dietary interventions—can substantially alter SBA synthesis, shifting the balance between pro-inflammatory and restorative mucosal phenotypes (25, 26). Consequently, targeting the microbiota–BA axis holds promise for reprogramming the immunosuppressive TME.

#### 3.1.3 Feedback regulation of BAs on gut microbiota

Conversely, BAs exert substantial feedback regulation on the gut microbiota, initiating a bidirectional crosstalk that markedly shapes the microbial landscape and influences tumor development (27, 28). Hydrophobic bile salts act as effective antimicrobial agents by disrupting bacterial cell membranes and inducing DNA damage, thereby selectively shaping microbial community structure based on BSH tolerance (29, 30).In genetically susceptible Apc^min/+^ murine models, excessive cholic acid depletes the anti-inflammatory genus *Lactobacillus* while enriching opportunistic pathobionts such as *Prevotella* and *Desulfovibrio*. This dysbiosis is associated with intestinal epithelial metaplasia (31).Similarly, DCA supplementation expands the relative abundance of *Parabacteroides* and *Bacteroides* while reducing BSH-producing *Lactobacillus* and *Clostridium* clusters XI/XIV (32). Ultimately, this BA-driven microbial imbalance can compromise epithelial barrier integrity and impair local immune surveillance, fostering a chronic inflammatory microenvironment that may facilitate favor tumor initiation and immune evasion.

#### 3.1.4 Role of BAs in tumor immune evasion and progression

BAs have been classically implicated in oncogenesis through oxidative stress and epithelial–mesenchymal transition (EMT) (33, 34), whereas accumulating evidence now positions them as central drivers of immune evasion within the TME (35, 36). In hepatocellular carcinoma (HCC), loss of FXR function leads to excessive intrahepatic BA accumulation, which may polarize tumor-associated macrophages toward an immunosuppressive M2 phenotype and thereby facilitate immune evasion (22, 37).

**Figure 2:**
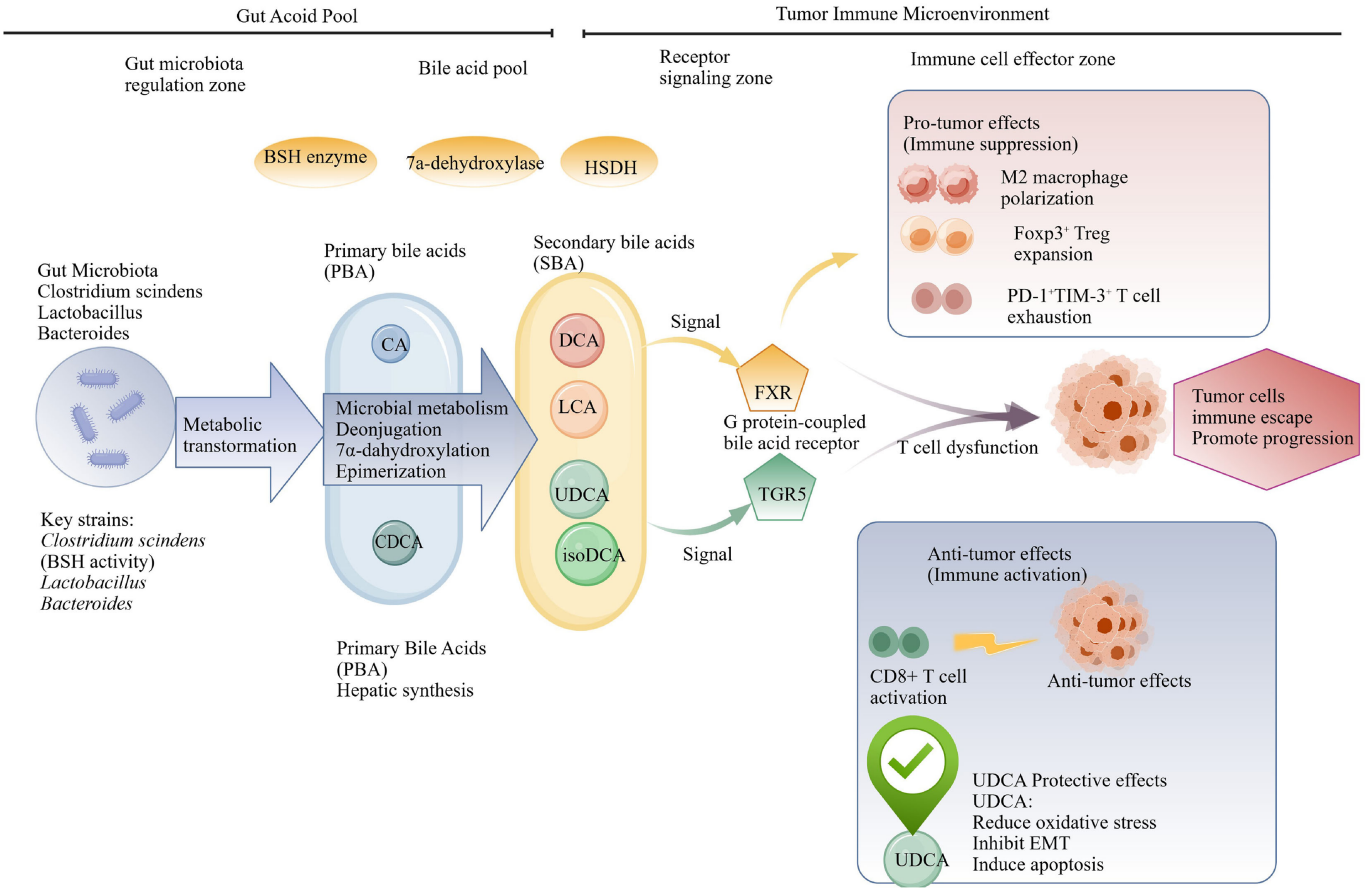
Gut microbiota-mediated bile acid metabolism and its dual immunomodulatory functions in tumor immune microenvironment. Primary bile acids synthesized in the liver are transformed into diverse secondary bile acids by intestinal bacteria and their metabolic enzymes. Distinct secondary bile acids activate FXR and TGR5 signaling pathways, exerting opposing pro-tumor and antitumor effects in the tumor microenvironment. (BSH, bile salt hydrolase; CA, cholic acid; CDCA, chenodeoxycholic acid; DCA, deoxycholic acid; EMT, epithelial-mesenchymal transition; FXR, farnesoid X receptor; HSDH, hydroxysteroid dehydrogenase; isoDCA, isodeoxycholic acid; LCA, lithocholic acid; PBA, primary bile acids; PD-1, programmed cell death protein 1; SBA, secondary bile acids; TIM-3, T cell immunoglobulin and mucin domain-containing protein 3; Treg, regulatory T cell; TGR5, Takeda G protein-coupled receptor 5; UDCA, ursodeoxycholic acid.).

Moreover, specific BA derivatives such as isolithocholic acid (isoDCA) can expand Foxp3^+^ regulatory T cells (Tregs) by modulating dendritic cell activation, thereby dampening antitumor immunity (17, 38). In light of these mechanisms, pharmacological reprogramming of BA metabolism has shown therapeutic promise. For example, BA sequestrants that lower systemic BA levels, or FXR agonists that counteract local inflammation, may help reverse T cell exhaustion and restore ICB efficacy (39). Thus, the capacity of BAs to modulate tumor immunity is correlated with their local concentrations. Beyond concentration, the effects of BAs on immunity also depend on the specific BA species involved. For instance, UDCA limits tumor progression by alleviating oxidative stress and inhibiting EMT (20, 21, 40) (**Figure 2**). Looking forward, characterizing the spatial distribution and species composition of BAs within the TME may facilitate prediction of ICB responses and guide microbiome-informed precision therapies.

### 3.2 TMAO

#### 3.2.1 The microbiota-liver metabolic axis

**Figure 3:**
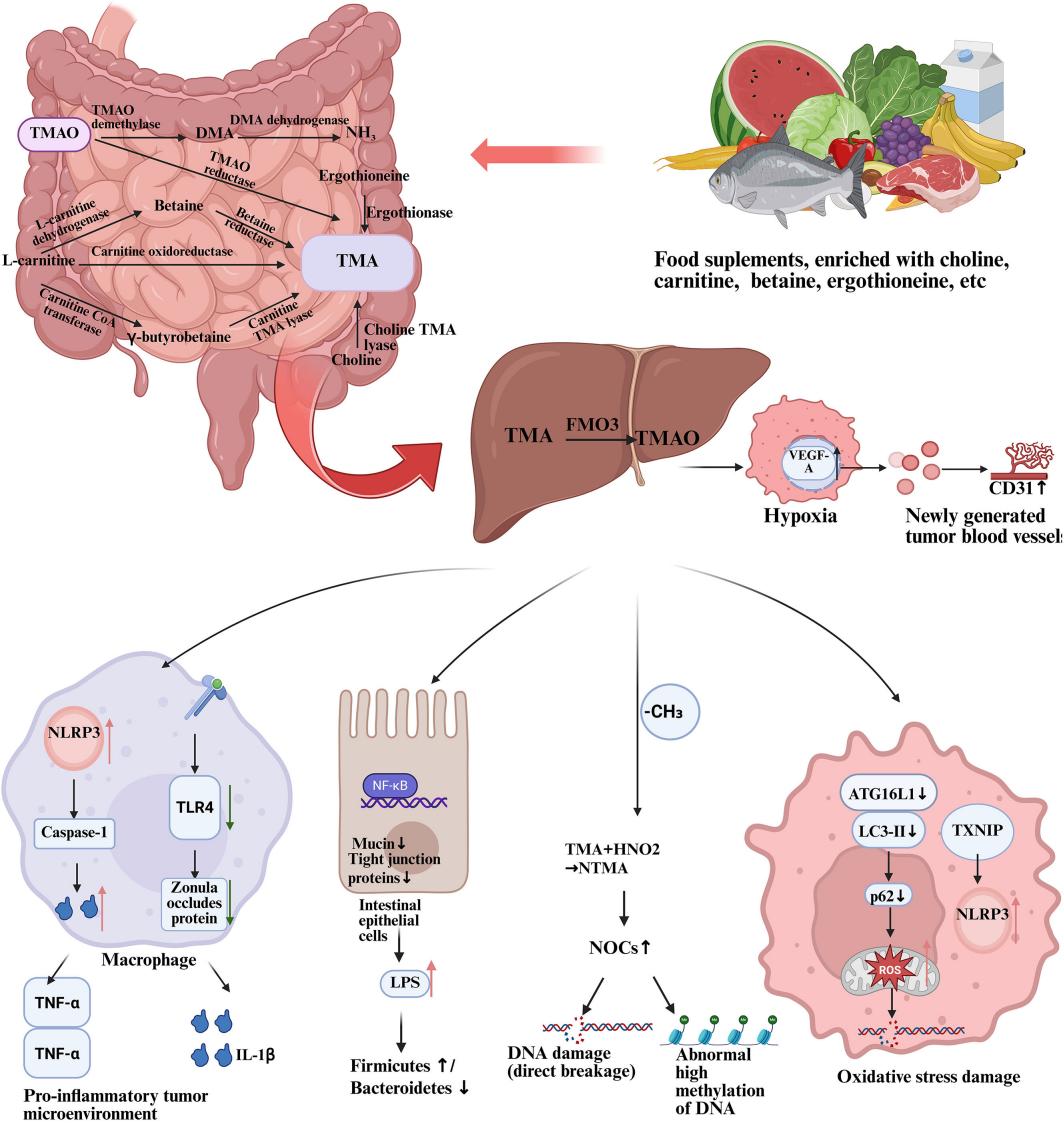
Core mechanistic Network of Trimethylamine N-Oxide (TMAO) in driving colorectal carcinogenesis. Dietary nutrients (e.g., choline, carnitine) are metabolized by gut microbiota into TMA, which is oxidized by hepatic FMO3 into TMAO. This axis promotes tumorigenesis through four primary mechanisms: (1) Angiogenesis: TMAO induces VEGF-A secretion and CD31 expression. (2) Inflammation: TMAO activates the NLRP3/Caspase-1 pathway in macrophages, increasing TNF-α and IL-1β. (3) Barrier Dysfunction: TMA reduces tight junction proteins, leading to LPS translocation and microbial dysbiosis. (4) Genotoxicity & Stress: TMA-derived N-nitroso compounds (NOCs) cause DNA damage, while inhibition of autophagy (ATG16L1/LC3-II) elevates ROS levels.(TMAO, Trimethylamine N-oxide; FMO3, Flavin Monooxygenase 3; NLRP3, NLR Family Pyrin Domain Containing 3; TMA, Trimethylamine; DMA, Dimethylamine; VEGF, Vascular Endothelial Growth Factor; CD31, Cluster of Differentiation 31; NF-κB, Nuclear Factor kappa-B; TLR4, Toll-like Receptor 4; LPS, Lipopolysaccharide; TNF-α, Tumor Necrosis Factor-alpha; IL-1β, Interleukin-1 beta; ROS, Reactive Oxygen Species; ATG16L1, Autophagy Related 16-Like 1; LC3-II, Microtubule-associated Protein 1A/1B-light chain 3-II; TXNIP, Thioredoxin-Interacting Protein; NOCs, N-nitroso Compounds).

TMAO is a metabolite generated through the combined action of the gut microbiota and hepatic enzyme systems. Commensal bacteria in the gut, such as *Firmicutes* harboring *CutC/D* enzymes, convert dietary precursors including choline and carnitine into trimethylamine (TMA) (41, 42) (**Figure 3**).TMA enters the portal circulation and is oxidized to TMAO in the liver by flavin monooxygenase 3 (FMO3) (41, 42). In addition, dietary TMAO (e.g., from seafood) can be directly absorbed by the intestine, whereas unabsorbed TMAO can be reduced back to TMA by certain bacteria via TMAO reductases in the gut, re-entering the liver for oxidation and forming an enterohepatic circulation (43). This metabolic axis is markedly dependent on the composition of the gut microbiota: administration of broad-spectrum antibiotics can substantially suppress TMAO production, which is restored after discontinuation, underscoring the dynamic dependence of TMAO on microbial homeostasis (44–46).

#### 3.2.2 Relationship between TMAO and the gut microbiota

A bidirectional regulatory relationship exists between TMAO and the gut microbiota. On one hand, the gut microbiota substantially influences TMAO production levels. Studies have shown that the ratio of *Firmicutes* to *Bacteroidetes* influences TMAO yield: when the *Firmicutes/Bacteroidetes* ratio is approximately 2:1, individuals tend to produce higher levels of TMAO, whereas a ratio of 1:1 is associated with low TMAO production (47).This is because *Firmicutes* harbor the enzymes required for converting choline to TMA, while *Bacteroidetes* lack the relevant enzymes (48). In addition, sex differences have been reported in TMAO production: in male mice, TMAO is primarily generated by *Clostridium* species in the gut, whereas in female mice, it appears to depend on *Ruminococcus* (49, 50). On the other hand, TMAO may also reshape the gut microbiota in a reciprocal manner. By activating oxidative stress and inflammatory signaling pathways, TMAO compromises intestinal barrier integrity and increases gut permeability, thereby promoting the expansion of opportunistic pathogens such as *Prevotella* and *Enterobacteriaceae*. This may establish a vicious cycle of “dysbiosis → barrier disruption → increased TMAO influx → exacerbated dysbiosis” (4, 47, 48). This bidirectional interplay forms the basis for understanding the dual role of TMAO in tumor immunity.

#### 3.2.3 Pro-tumor mechanisms of TMAO: insights from colorectal cancer

In colorectal cancer (CRC), TMAO has been reported to undermine the efficacy of immunotherapy, at least in part by remodeling the immunosuppressive TME. First, TMAO activates the NLRP3 inflammasome and induces oxidative stress, which promotes the polarization of tumor-associated macrophages (TAMs) toward an immunosuppressive M2 phenotype, thereby suppressing the activation and infiltration of CD8^+^ T cells (51, 52). Second, TMAO has been shown to upregulate VEGFA signaling and may promote aberrant angiogenesis, and form a physical barrier that impedes the entry of effector T cells into the tumor parenchyma, thus diminishing the efficacy of immune checkpoint inhibitors (e.g., anti-PD-1) (53–55).

Moreover, TMAO has been found to disrupt intestinal barrier integrity, leading to the translocation of bacterial products into the circulation and triggering systemic low-grade inflammation, which may further expand regulatory T cells (Tregs) and establish an immunosuppressive milieu (56–58).

#### 3.2.4 TMAO as a sensitizer for antitumor immunity and immune checkpoint blockade

In contrast to its pro-tumorigenic role in CRC, TMAO appears to exhibit a distinct function in enhancing antitumor immunity in non-gastrointestinal tumors such as pancreatic ductal adenocarcinoma (PDAC). Studies have shown that in PDAC models, exogenous TMAO polarizes tumor-associated macrophages (TAMs) toward an immunostimulatory phenotype rather than an immunosuppressive one (4). Mechanistically, TMAO has been shown to amplify type I interferon signaling and activate the endoplasmic reticulum stress kinase PERK in malignant cells, inducing immunogenic cell death in this model (56). This process has been shown to enhance the infiltration and effector function of CD8^+^ T cells, reprogramming the “cold” tumor microenvironment into a “hot” one, thereby exhibiting synergy with immune checkpoint inhibitors (e.g., anti−PD−1, anti−Tim−3) to reduce tumor burden and prolong survival (4, 56–58).

Notably, although TMAO has been shown to act as an immunotherapeutic sensitizer in both PDAC and triple−negative breast cancer (TNBC), the specific molecular mechanisms by which it induces tumor cell death appear to differ between these cancer types. In TNBC models, a study by Wang et al. demonstrated that TMAO induces GSDME−dependent pyroptosis through activation of the PERK signaling axis, rather than generalized immunogenic cell death (59). This pyroptotic process releases substantial damage-associated molecular patterns (DAMPs) and pro-inflammatory cytokines, thereby recruiting and activating antigen−presenting cells and initiating antitumor T-cell immune responses. This finding provides a parallel to the pro−tumorigenic mechanism described in Section 3.2.3 of this review, where TMAO activates the NLRP3 inflammasome and induces non−specific inflammation in colorectal cancer: the regulation of the inflammasome–gasdermin pathway (NLRP3 inflammasome and downstream GSDME/GSDMD) by TMAO appears to be context-dependent—driving immunosuppression via the NLRP3 inflammasome in CRC, while triggering pyroptosis through the PERK–GSDME axis in TNBC, thereby generating protective antitumor immunity.

Current evidence regarding the immunomodulatory role of TMAO remains controversial. As summarized in a recent review, multiple observational studies have shown that TMAO has been associated with chronic inflammation through activation of NF−κB and the NLRP3 inflammasome, and its levels have been positively associated with the risk of cardiovascular disease, metabolic disorders, and multiple cancers (59). However, Wang et al. in TNBC models and Mirji et al. in PDAC models found that TMAO has been reported to suppress tumor progression and enhance responses to immunotherapy (56, 59). This discrepancy reflects the context−dependent nature of TMAO as a “microbiome–host metabolic messenger”: its immunological effects appear to depend on tumor type, TMAO concentration, local microenvironment, and microbial context (59). In particular, TMAO drives immunosuppressive M2 macrophage polarization through the NLRP3 inflammasome in CRC, yet activates antitumor immunity via the PERK–GSDME pyroptosis pathway in TNBC—this contrast indicates that the net effect of TMAO may be markedly dependent on tissue−specific immune microenvironments and differential downstream cell death pathways. Notably, in the context of cardiovascular disease, the study by Saaoud et al. has further shown that TMAO activates PERK and its downstream ER stress/mitochondrial ROS/glycolysis pathways, thereby inducing trained immunity in macrophages and contributing to atherosclerotic inflammation (57). This finding provides clues for understanding the differential effects of TMAO via the PERK axis across distinct tissues: PERK activation has been reported to trigger antitumor immunity in TNBC and PDAC, whereas in the cardiovascular system, PERK activation has been shown to promote pro-inflammatory trained immunity, highlighting the context-dependent nature of the TMAO–PERK signaling axis (57).Currently, the direct receptor for TMAO has not been identified, and the molecular mechanisms underlying its tissue-specific effects remain to be fully elucidated.

#### 3.2.5 Clinical translation: TMAO as a predictive biomarker and therapeutic target

The above findings suggest that TMAO may have clinical translational value. First, retrospective analyses in patients with pancreatic ductal adenocarcinoma, melanoma, and triple-negative breast cancer have reported that higher circulating TMAO levels or the presence of TMA-producing bacteria (e.g., microbiota harboring the *CutC* gene) are associated with better responses to immune checkpoint inhibitors and longer survival (4, 56–58).Thus, circulating TMAO has been considered a potential biomarker for predicting immunotherapy efficacy. Second, intervention strategies targeting TMAO should take into consideration its tumor type-dependent effects. In tumors where TMAO exerts pro-tumorigenic effects (e.g., colorectal cancer), preclinical studies suggest that TMAO levels may be reduced through restriction of dietary precursor intake, use of FMO3 inhibitors, reactive oxygen species scavengers, or NLRP3 inflammasome inhibitors to modulate the immunosuppressive TME (51, 52).However, in pancreatic ductal adenocarcinoma, melanoma, and triple-negative breast cancer, TMAO has been shown to act as an immunostimulant and ICB sensitizer in animal models (56–58). The above strategies aimed at lowering TMAO may produce opposite effects in these contexts, potentially undermining antitumor immunity. Therefore, future translational strategies should be differentially designed based on tumor type: targeted degradation or inhibition of TMAO synthesis in tumors where TMAO appears to be detrimental, and supplementation with TMAO-producing probiotics or their precursors (e.g., choline) in tumors where TMAO appears to be beneficial, to maintain or enhance local immune responses. This precision intervention approach, grounded in the metabolic characteristics of the tumor immune microenvironment, represents a prerequisite for the clinical translation of TMAO-targeted therapies.

Currently, clinical translation of TMAO-targeted pathways remains in its early stages. In the field of tumor immunotherapy, the only directly relevant registered clinical trial is the ongoing Phase II Renaissance study (Fudan University), which aims to evaluate the efficacy of oral choline combined with anti-PD-1 antibody in triple-negative breast cancer (60). In the domain of dietary and lifestyle interventions, a systematic review published in 2025 evaluated 27 clinical trials and found that plant-based diets, high-dairy diets, very-low-calorie ketogenic diets, and Mediterranean diets were associated with lower TMAO levels, whereas high-protein and high-fat diets were associated with higher TMAO levels (61). However, the relationship between dietary interventions and TMAO remains controversial, with some study results conflicting, indicating that more rigorously designed RCTs are needed for verification. In addition, a systematic review of 13 RCTs (total n=553) examining the relationship between red meat consumption and TMAO found that the effects of red meat intake on TMAO levels were inconsistent: when red meat intake was high (71–420 g/day), most studies observed TMAO elevation; however, when intake was low (60–156 g/day), no significant difference was detected (62). The authors concluded that current evidence is insufficient to support a causal relationship between red meat intake and TMAO levels, and further high-quality studies are required. In the cardiovascular field, although animal studies have shown that TMAO lyase inhibitors and antisense oligonucleotide (ASO) therapies can reduce TMAO levels and attenuate atherosclerosis, these TMAO-targeting interventions have not yet entered human clinical trials (63). Currently, indirect strategies (e.g., dietary modification, probiotic interventions) are clinically feasible approaches for lowering TMAO levels. In summary, the clinical application of TMAO in tumor immunotherapy remains in its early stages.

### 3.3 SCFAs

#### 3.3.1 SCFA metabolism and epigenetic immunomodulation

SCFAs—primarily acetate, propionate, and butyrate—are anionic metabolites derived from the colonic microbial fermentation of dietary fibers (64). Beyond their conventional role as an energy source for colonic epithelial cells, these metabolites traverse the epithelium into the systemic circulation, functioning as regulators of host immunity across multiple organ systems (65, 66). In the context of oncology, their relevance lies in their capacity to maintain tight junction integrity and suppress chronic mucosal inflammation—two firewalls against tumor initiation. Specific SCFA species exhibit distinct protective profiles; for instance, butyrate has been reported to alleviate local oxidative stress, while propionate has been shown to suppress pathogenic bacterial overgrowth and modulate local inflammatory cascades (66–68). At the molecular level, SCFAs are thought to coordinate these immunomodulatory effects within the TME through two conserved mechanistic axes. First, they serve as natural ligands for cell-surface G protein-coupled receptors (primarily GPR41 and GPR43), which are expressed on various innate and adaptive immune cell populations. Second, and notably for tumor immunology, SCFAs—particularly butyrate—act as inhibitors of histone deacetylases (HDACs) (69). This HDAC inhibition has been associated with epigenetic reprogramming within the TME, influencing chromatin accessibility and contributing to the differentiation of regulatory subsets and the functional polarization of tumor-infiltrating myeloid cells. Furthermore, by acting as acetyl-CoA precursors, SCFAs may contribute to the metabolic and transcriptional rewiring necessary for antitumor immunity (69).

However, it should be noted that the immunomodulatory effects of SCFAs are not uniformly anti-tumorigenic or protective across all contexts. Recent studies have revealed the dual nature of SCFA functions, with butyrate being a representative example. In viral infection models, although butyrate can attenuate influenza virus-induced lung injury by inducing macrophage polarization toward an alternatively activated phenotype through FFAR3, it has also been found to suppress the expression of interferon-stimulated genes (ISGs) such as RIG-I and IFITM3, thereby promoting the infection and replication of influenza virus, HIV-1, and human metapneumovirus. This modulation of antiviral immunity may exert complex effects in the context of tumor immunotherapy—if patients develop viral infections during immunotherapy, SCFA-mediated ISG suppression could interfere with antiviral immune responses, thereby indirectly affecting overall treatment tolerance. Furthermore, the regulation of interferon signaling by SCFAs appears to be species-dependent: acetate enhances IFN-β production through a GPR43-dependent pathway to inhibit respiratory syncytial virus, whereas butyrate suppresses type I IFN signaling (70). This functional heterogeneity suggests that the net effect of SCFAs within the tumor microenvironment may depend on the specific SCFA composition profile, tumor type, and host immune status, and that labeling them as “antitumor metabolites” risks oversimplification. Notably, among the major SCFAs, propionate and acetate have exhibited more consistently anti-tumorigenic profiles across the diverse contexts studied to date, whereas butyrate displays a pronounced context-dependent duality—acting as an immunotherapy sensitizer in some settings while having been associated with radioresistance and potential impairing antiviral immunity in others.

#### 3.3.2 Microbiota-SCFA crosstalk in shaping barrier immunity

Short-chain fatty acids are primarily synthesized through the microbial fermentation of indigestible dietary fibers, such as pectin and inulin (69).This biosynthetic capacity is partitioned among specialized commensal consortia: acetate is primarily produced by the genera *Bacteroides* and *Bifidobacterium*, propionate by *Prevotella* and *Roseburia*, and butyrate by *Faecalibacterium* and *Eubacterium*. Rather than acting as passive metabolic byproducts, SCFAs influence the intestinal microecosystem. By releasing protons, they can lower the luminal pH to establish an environment that can inhibit the expansion of opportunistic pathogens while fostering the colonization of beneficial microbes. Consequently, targeted dietary interventions utilizing specific prebiotics (e.g., inulin) can selectively enrich butyrate-producing taxa, thereby influencing the overall microbial architecture to support local immune surveillance (71).

**Figure 4:**
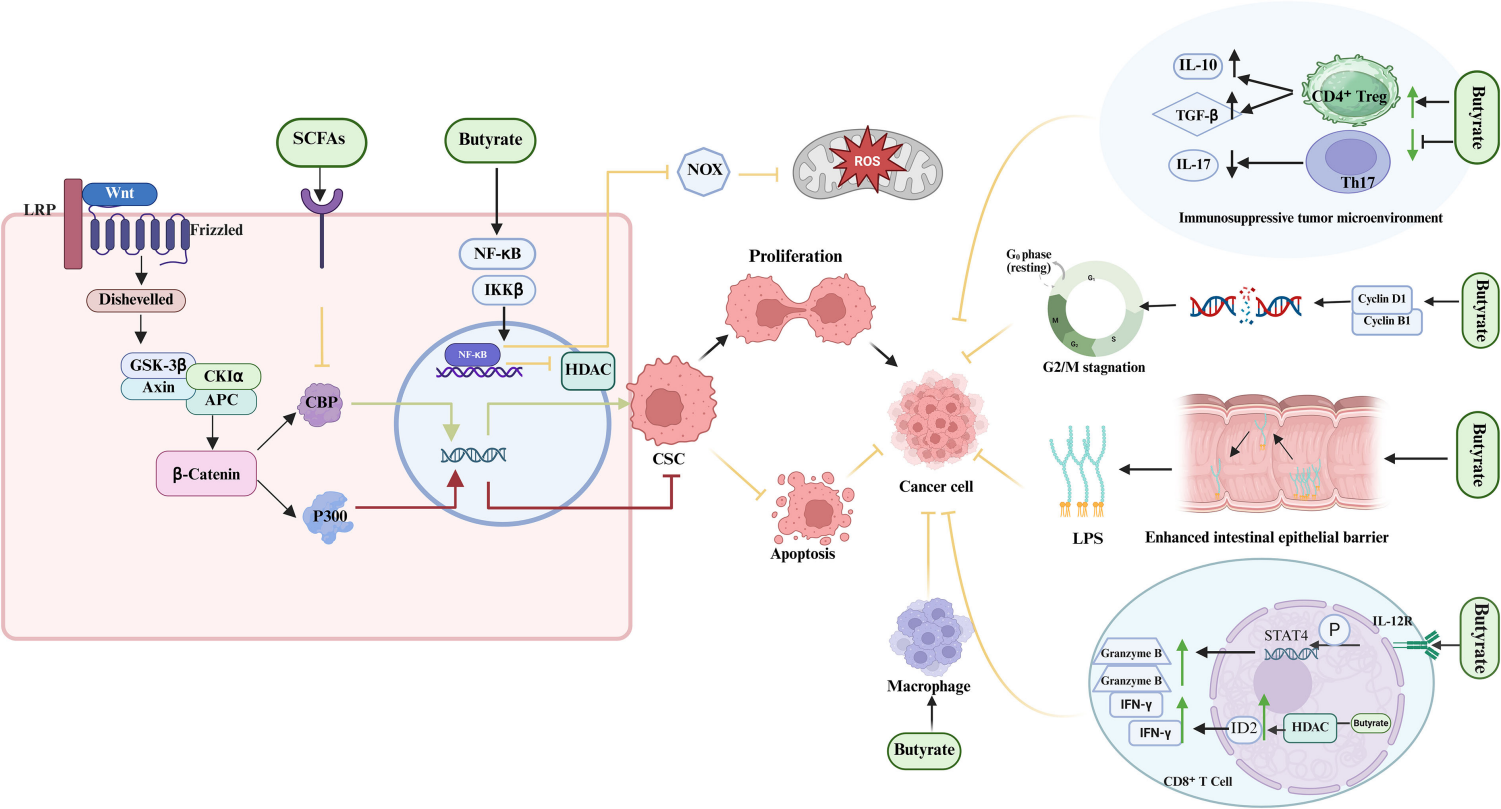
Mechanism of the antitumor effect of butyrate. SCFAs, particularly butyrate, exert antitumor effects through multiple regulatory axes:Intracellular Signaling: Butyrate inhibits HDAC activity, leading to the suppression of the NF-κB/IKKβ pathway and antagonism of Wnt/β-catenin signaling via CBP/P300 modulation. This combined action inhibits cancer stem cell (CSC) properties and tumor proliferation.Cell Cycle and Apoptosis: Butyrate induces G2/M phase stagnation by downregulating Cyclin D1/B1 and promotes cancer cell apoptosis while reducing oxidative stress by inhibiting NOX-mediated ROS production.Immune Modulation: Butyrate suppresses the immunosuppressive TME by inhibiting Treg expansion (reducing IL-10 and TGF-β) and Th17 differentiation. Conversely, it enhances the effector function of CD8^+^ T cells by promoting IFN-γ and Granzyme B production through the STAT4 and ID2 pathways.Barrier Maintenance: Butyrate strengthens the intestinal epithelial barrier, preventing LPS translocation and subsequent systemic inflammation.(SCFAs, Short-Chain Fatty Acids; HDAC, Histone Deacetylase; GPR41/43, G Protein-Coupled Receptor 41/43; NF-κB, Nuclear Factor kappa-B; IKKβ, IκB Kinase beta; GSK-3β, Glycogen Synthase Kinase-3 beta; APC, Adenomatous Polyposis Coli; CBP, CREB-binding Protein; P300, E1A-binding protein P300; CSC, Cancer Stem Cell; ROS, Reactive Oxygen Species; NOX, NADPH Oxidase; Treg, Regulatory T cell; Th17, T helper 17 cell; IL-10, Interleukin-10; TGF-β, Transforming Growth Factor-beta; IL-17, Interleukin-17; STAT4, Signal Transducer and Activator of Transcription 4; ID2, Inhibitor of DNA Binding 2; IFN-γ, Interferon-gamma; IL-12R, Interleukin-12 Receptor; LPS, Lipopolysaccharide).

This SCFA-microbiota crosstalk has been shown to reinforce the intestinal barrier against dysbiosis-driven oncogenesis. SCFAs engage GPR41 and GPR43 on the intestinal epithelium to stimulate the secretion of antimicrobial peptides from Paneth cells and mucins from goblet cells, contributing to the maintenance of the mucosal barrier (72). Within the lamina propria, these metabolites influence immunoregulatory processes. By binding to GPR43 on localized Th1 cells, SCFAs have been reported to activate the STAT3/mTOR signaling cascade, which has been associated with upregulation of the anti-inflammatory cytokine IL-10.signaling (73) (**Figure 4**). This signaling axis has been shown to modulate chronic mucosal inflammation, potentially reducing the establishment of a pro-tumorigenic microenvironment that may contribute to colorectal malignancies.

#### 3.3.3 SCFA-driven reprogramming of the tumor immune microenvironment

Short-chain fatty acids can influence the localized immunological landscape of the TME by modulating innate and adaptive cellular compartments. Butyrate has been reported to mediate the functional maturation of macrophages and modulate local inflammation by expanding CD4^+^ regulatory T cells (Tregs) via GPR43 engagement while influencing Th17-mediated secretion of pro-inflammatory cytokines (IL-6, TNF-α) (74). In the context of tumor immune evasion, butyrate has been shown to stimulate the IL-12 signaling axis, leading to the marked upregulation of the transcriptional regulator ID2. This IL-12-ID2 cascade has been associated with enhanced secretion of IFN-γ and granzyme B, supporting the cytolytic capacity of tumor-infiltrating CD8^+^ T cells (75). Acetate has been reported to complement this process by downregulating the PVR/CD155 axis on malignant cells and inhibiting the PI3K/AKT pathway, which may rescue CD8^+^ T cells from immunosuppressive states (76). Concurrently, propionate has been shown to augment natural killer (NK) cell cytotoxicity via the mTORC2/PDK1/AKT signaling cascade and modulate pro-oncogenic cytokine secretion from γδ T cells, contributing to a localized immune barrier (53, 77, 78).

#### 3.3.4 Epigenetic restraint and systemic immune surveillance

Beyond local cellular interactions, SCFAs have been reported to exert tumor-suppressive effects through systemic epigenetic regulation and barrier maintenance. Butyrate acts as an histone deacetylase (HDAC) inhibitor. This epigenetic function has been associated with modulation of oncogenic NF-κB signaling—a frequently hyperactive cascade in colorectal cancers—while altering chromatin accessibility and reinforcing the intestinal barrier to limit systemic LPS translocation (5, 78, 79) (**Figure 4**). Propionate has been reported to synergize with this epigenetic defense by promoting the degradation of the silencing enzyme EHMT2 (80). On a systemic level, these metabolites may bridge distant organ networks. For instance, the restoration of systemic acetate pools (driven by specific commensals like *Lactobacillus reuteri*) has been shown to facilitate retinoic acid-mediated IgA class switching in B cells to support mucosal defenses (81, 82). Acetate administration has been reported to reverse chronic stress-induced immunosuppression, thereby inhibiting stress-accelerated tumor progression via the gut-brain-tumor axis (83).

#### 3.3.5 Therapeutic synergy and the radiosensitization dichotomy

The clinical utility of optimizing SCFA pools may be contingent on the targeted therapeutic modality. Specific SCFAs act as sensitizers for advanced therapies: acetate has been shown to enhance the efficacy of photodynamic therapy (PDT) by increasing reactive oxygen species (ROS) generation (84), while exogenous butyrate and propionate have been shown to synergize with chemotherapeutics like oxaliplatin and cisplatin to modulate epithelial-mesenchymal transition (EMT) and promote tumor apoptosis (75, 85, 86). Furthermore, optimizing systemic propionate and acetate levels via interventions such as FMT has been reported to bolster the efficacy of immune checkpoint blockade (e.g., anti-PD-1) (87). Complicating this therapeutic landscape, however, butyrate displays a radiosensitization dichotomy. It has been shown to suppress the STING-Type I interferon signaling pathway within dendritic cells, thereby modulating ionizing radiation-induced immunity. Consequently, targeted depletion of butyrate-producing bacteria has been reported to sensitize tumors to radiotherapy (88, 89), underscoring the need for context-specific microbiome interventions.

#### 3.3.6 Clinical translation progress

The aforementioned mechanisms are being advanced toward clinical translation, currently encompassing three main strategies: high-fiber dietary patterns and Mediterranean diets have been shown to increase the abundance of SCFA-producing microbiota and have been associated with prolonged progression-free survival in immunotherapy in observational studies (90); Fecal microbiota transplantation (FMT) has been reported to achieve objective response rates of 20–40% in small−cohort pilot studies of melanoma patients resistant to immune checkpoint inhibitors (91); and supplementation strategies with butyrate-producing bacteria such as *Faecalibacterium prausnitzii* are under clinical development. However, this field still faces challenges: the dual nature of SCFAs suggests that indiscriminately elevating their levels may be counterproductive, and off-label probiotic overuse has been associated with poor prognosis, highlighting the need for patient stratification strategies based on microbiome analysis and standardized intervention protocols.

The immunomodulatory effects of the aforementioned microbial metabolites are integrated and amplified through the host cell’s own metabolic networks. Among these, one-carbon metabolism, as a central hub connecting nutrient sensing, epigenetic modification, and immune effector functions, plays a role in this integration process. Jiang et al. systematically reviewed the multifaceted functions of one-carbon metabolism in cancer: providing one-carbon units for nucleotide synthesis, regulating histone and DNA methylation, maintaining NADPH/GSH homeostasis to counteract oxidative stress, and modulating the functional differentiation of T cells and macrophages (92). Gut microbial metabolites (such as folate, vitamin B12, serine, and glycine) as well as acetyl-CoA derived from SCFA metabolism can serve as substrates or cofactors for one-carbon metabolism, potentially influencing the epigenetic status and effector functions of immune cells within the TME (92). Therefore, integrative analysis of microbial metabolites and host one-carbon metabolism may offer insights into the regulatory mechanism of “diet–microbiota–metabolism–epigenetics–immunity.”

### 3.4 Recent research hotspots: immunological mechanisms of gut metabolites in the TME

Studies examining the interface between gut microbial metabolites and tumor immunity have demonstrated that these molecules function as signaling factors within the TME. Beyond the three major classes of classical metabolites discussed above, a range of novel microbial metabolites have also emerged in recent years with immunomodulatory functions. The regulation of tumor immunity by the microbiota is not limited to the direct action of metabolites on immune cells, but also involves broader reprogramming of innate immune cells within the TME, particularly macrophages. A recent single-cell transcriptomic study by Cao et al. revealed this dimension: the microbiota can reprogram SPP1^+^ tumor-associated macrophages (TAMs) into CD74^+^ antigen-presenting cells, which in turn activate a γδ T cells–antigen-presenting cell–CD8^+^ T cell immune cascade through the CD40L–CD40/NF-κB signaling axis (93). This finding suggests that the aforementioned microbial metabolites (such as LPS, IPA, and SCFAs) may serve as molecular mediators of this intercellular interaction network by inducing phenotypic and functional reprogramming of TAMs. In the following sections, we summarize how specific microbial metabolites regulate T cell exhaustion, influence the accumulation of immunosuppressive myeloid cells, and modulate lymphoid structures, following similar mechanistic principles.

#### 3.4.1 Modulation of CD8^+^ T cell exhaustion and persistence

Gut metabolites exert context-dependent effects on the effector function and exhaustion trajectory of CD8^+^ T cells. The microbiota-derived metabolite PAGln, primarily synthesized by *Enterococcus faecalis*, has been reported to disrupt T-cell activity. Elevated serum PAGln concentrations correlate with resistance to anti-PD-1 therapy. Mechanistically, PAGln has been shown to suppress the secretion of effector cytokines, including IFN-γ and TNF-α, by CD8^+^ T cells. This suppression has been associated with T-cell exhaustion and may impair antitumor immune responses (94).

Other metabolites preserve or enhance T-cell function. Urolithin A has been shown to amplify CD8^+^ T-cell-mediated antitumor responses by targeting the ERK1/2-ULK1 molecular axis. This compound has been reported to induce mitochondrial autophagy and adaptive metabolic remodeling, potentially maintaining reactive oxygen species (ROS) homeostasis and supporting the functional persistence of primary CD8^+^ T cells and CAR-T cells (95). IPA, produced synergistically by *Lactobacillus johnsonii* and *Clostridium sporogenes*, has been reported to maintain CD8^+^ T cell stemness. By increasing H3K27 acetylation in the superenhancer region of the *Tcf7* gene, IPA has been shown to promote the differentiation of progenitor-like exhausted CD8^+^ T cells and synergize with anti-PD-1 immunotherapy (96).

#### 3.4.2 Accumulation of MDSCs and polarization of TAMs

The expansion of immunosuppressive myeloid cells, including myeloid-derived suppressor cells (MDSCs) and tumor-associated macrophages (TAMs), may be driven by gut metabolites. The microbial metabolite 4-hydroxyphenylacetic acid (4-HPA) has been reported to recruit PMN-MDSCs to the colorectal cancer microenvironment. 4-HPA has been shown to activate the JAK2/STAT3 signaling pathway to upregulate CXCL3 expression, leading to immunosuppression that may compromise the efficacy of PD-1 blockade (97).

Arginine-derived polyamines also shape the TAM compartment. Gut microbiota-derived arginine precursors are reprogrammed by tumor cells into polyamines such as spermidine, which have been reported to induce thymine DNA glycosylase (TDG)-mediated DNA demethylation. This epigenetic shift has been associated with polarization of TAMs toward a protumoral phenotype. Disrupting the arginine-polyamine-TDG axis has been shown to reverse this immunosuppressive state and restore the antitumor activity of tumor-infiltrating lymphocytes (TILs) (98).Beyond the myeloid suppression mediated by the microbial metabolites discussed above, the intrinsic hypoxia–metabolic signaling pathway in TAMs also plays a role in immunotherapy resistance and may synergize with microbial metabolites to produce additional effects. Hou et al. reported that under hypoxic conditions, elevated HIF-1α expression in TAMs has been associated with the release of VEGF-rich exosomes (exoVEGF), which may drive tumor angiogenesis through NF-κB, PI3K/AKT, and p38/MAPK signaling pathways, thereby contributing to immunotherapy resistance (99). As noted earlier in this review, microbial metabolites such as LPS and TMAO have been shown to activate the NF-κB pathway, while SCFAs can modulate PI3K/AKT signaling via GPCRs. Therefore, it is plausible that microbial metabolites and the intrinsic HIF-1α signaling in TAMs may act in concert to influence the immunosuppressive microenvironment. This crosstalk offers a therapeutic rationale for combined targeting of microbial metabolites and hypoxia signaling pathways.

#### 3.4.3 Modulation of Tregs and tertiary lymphoid structures via the AhR axis

Dietary tryptophan metabolites modulate regulatory T cells (Tregs) and the spatial organization of immune cells primarily through the AhR signaling pathway. Pathogenic bacteria such as *Klebsiella* metabolize tryptophan into kynurenine. Subsequent AhR activation by kynurenine has been reported to induce pro-inflammatory Th17 cells and contribute to an immune-privileged TME by modulating effector Tregs (100). The accumulation of kynurenine also depletes local tryptophan, an amino acid essential for effector T-cell proliferation, and has been associated with impaired maturation of tertiary lymphoid structures (TLSs). Inhibiting this metabolic pathway has been shown to reverse local immunosuppression and enhance the activation of follicular helper T cells and B cells within TLSs (101).

Alternatively, IPA acts as an endogenous ligand for both AhR and the pregnane X receptor (PXR) to initiate anti-inflammatory signaling. This receptor activation has been reported to regulate the balance between Tregs and proinflammatory Th17 cells, modulating systemic inflammation and influencing the TME with respect to a cancer-promoting inflammatory state (8, 102).

## 4 Microbe-associated molecular patterns – lipopolysaccharide

### 4.1 Metabolism and functions of LPSs

LPS, the principal endotoxin derived from the outer membrane of Gram-negative bacteria, exerts substantial immunomodulatory effects that are largely shaped by the structural configuration of its lipid A moiety (103, 104).

**Figure 5:**
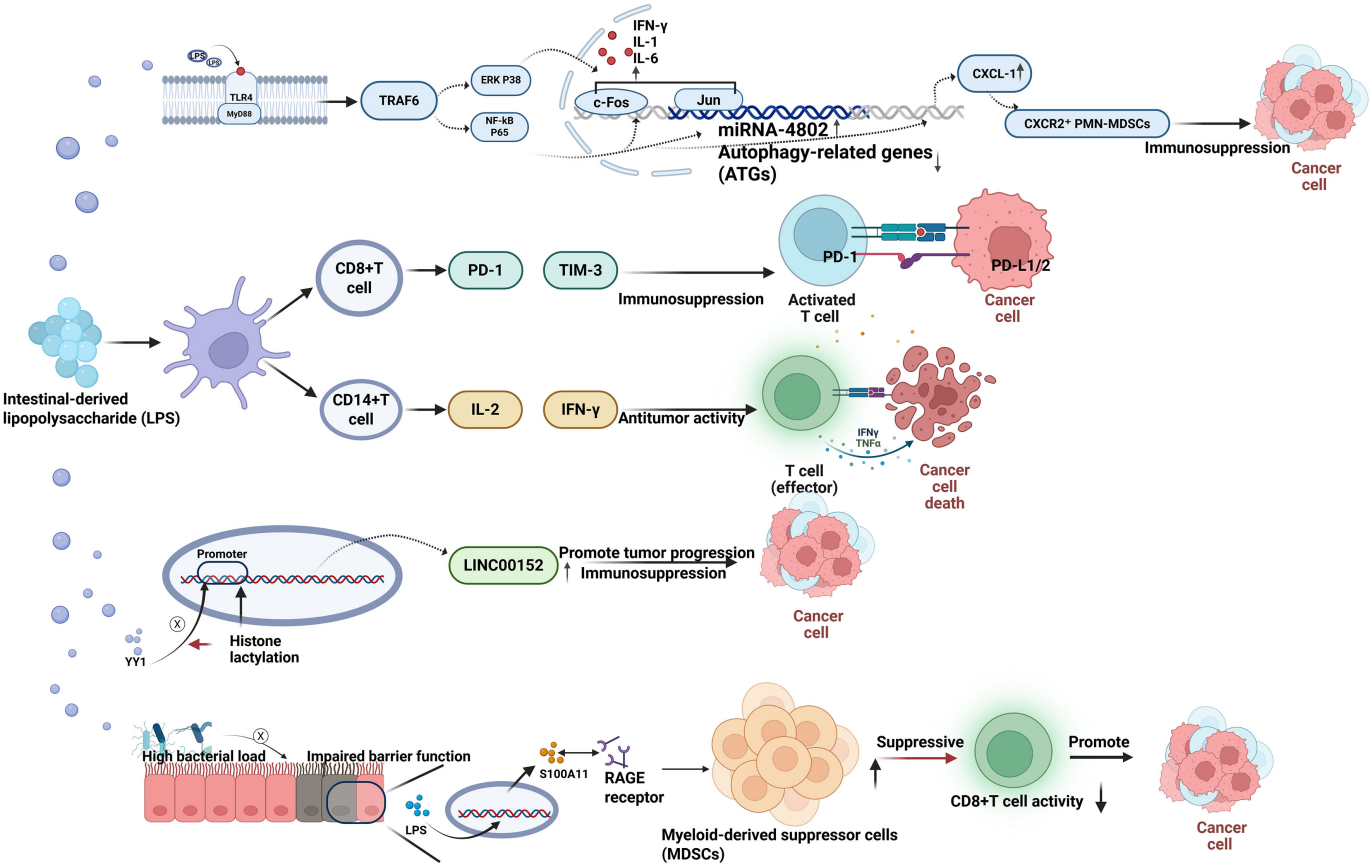
The Impact of Lipopolysaccharide (LPS) on tumor immunity and related molecular mechanisms. Impaired intestinal barrier function leads to the translocation of LPS, which drives tumor progression through multiple immunological and epigenetic pathways. Translocated LPS activates the TLR4/MyD88 signaling axis, signaling through TRAF6 to trigger ERK/P38 and NF-κB/P65 cascades. This pathway promotes the transcription of miRNA-4802 and downregulates autophagy-related genes (ATGs), leading to the secretion of CXCL-1 and subsequent recruitment of CXCR2^+^ PMN-MDSCs, which establish a robust immunosuppressive environment. LPS differentially modulates T cell polarization: it induces the expression of exhaustion markers PD-1 and TIM-3 on CD8^+^ T cells, facilitating interaction with PD-L1/2 on cancer cells to cause immune paralysis; simultaneously, it impairs the production of IL-2 and IFN-γ in CD14^+^ T cells, suppressing antitumor activity. On an epigenetic level, LPS triggers histone lactylation and YY1 recruitment to activate the long non-coding RNA LINC00152, directly promoting malignancy. Additionally, high bacterial loads secondary to barrier failure activate MDSCs via the S100A11/RAGE receptor axis, further inhibiting CD8^+^ T cell activity and accelerating tumor growth. (ATGs, autophagy-related genes; CD8, cluster of differentiation 8; CD14, cluster of differentiation 14; CXCL-1, C-X-C motif chemokine ligand 1; CXCR2, C-X-C motif chemokine receptor 2; ERK, extracellular regulated protein kinase; IFN-γ, interferon-gamma; IL-1, interleukin-1; IL-2, interleukin-2; IL-6, interleukin-6; LINC00152, long intergenic non-coding RNA 00152; LPS, lipopolysaccharide; MDSCs, myeloid-derived suppressor cells; miRNA, microRNA; MyD88, myeloid differentiation primary response 88; NF-κB, nuclear factor kappa-light-chain-enhancer of activated B cells; PD-1, programmed cell death protein 1; PD-L1/2, programmed death ligand 1/2; PMN-MDSCs, polymorphonuclear myeloid-derived suppressor cells; P38, P38 mitogen-activated protein kinase; P65, NF-κB p65 subunit (RelA); RAGE, receptor for advanced glycation end products; S100A11, S100 calcium-binding protein A11; TIM-3, T cell immunoglobulin and mucin domain-containing protein 3; TLR4, Toll-like receptor 4; TNFα, tumor necrosis factor-alpha; TRAF6, tumor necrosis factor receptor-associated factor 6; YY1, Yin Yang 1 transcription factor; c-Fos, cellular Fos proto-oncogene; Jun, Jun proto-oncogene.).

Upon translocation through a compromised intestinal barrier, LPS forms a complex with lipopolysaccharide-binding protein (LBP) and engages the CD14/TLR4/MD2 receptor complex on myeloid cells, such as monocytes and macrophages. This interaction triggers pronounced NF-κB-mediated inflammatory cascades that can disrupt mucosal homeostasis and are linked to the development and progression of CRC (105, 106) (**Figure 5**).

Systemically, hepatic Kupffer cells act as the primary clearance mechanism for translocated LPS, internalizing and enzymatically degrading the highly reactive lipid A into inert derivatives. The immunological consequences of LPS exposure within the TME are highly species-dependent. While pathogenic LPS typically provokes pro-tumorigenic inflammation, commensal-derived variants often foster immune tolerance. For instance, a structurally distinct Lipid A variant (4A-MPLA) produced by the commensal Bacteroides fragilis induces a sustained IFN-β response in dendritic cells. Rather than fueling systemic inflammation, this signaling axis enhances the suppressive function of Tregs while dampening pathogenic Th17 responses via the type I interferon receptor (IFNAR), thereby modulating the immune balance within the local microenvironment (107, 108).

### 4.2 Microbiota-driven modulation of LPS translocation and bioactivity

The dynamic interplay between the gut microbiota and the intestinal mucosal barrier influences the systemic burden of LPS. Dysbiosis-induced disruption of the epithelial barrier—often characterized by a concurrent reduction in beneficial metabolites and the degradation of critical tight junction proteins such as ZO-1 and occludin—can lead to a “leaky gut” phenotype. This increased permeability substantially enhances systemic LPS translocation (109) (**Figure 5**). Rather than merely inducing localized inflammatory liver injury, this chronic endotoxin influx establishes a systemic pro-inflammatory baseline. Within the hepatic niche, persistent activation of TLR4/NF-κB signaling by translocated LPS can create an inflamed yet immunosuppressive microenvironment. This metabolic-immune crosstalk may paradoxically accelerates the progression of hepatocellular carcinoma (HCC) and provide a fertile pre-metastatic niche for distant extra-intestinal tumors.

Furthermore, the resident microbiota shapes the immunogenicity of the LPS pool. While endotoxins derived from opportunistic pathogens like Escherichia coli drive the secretion of pro-tumorigenic cytokines (e.g., TNF-α and IL-6), commensal-derived variants tend to exert protective, immunoregulatory effects (110). Consequently, therapeutic modulation of the microbiome offers a strategic avenue to alter this endotoxin profile. For example, dietary interventions with isoflavone-rich regimens have been shown to reshape microbial composition and attenuate the biosynthesis of immunogenic LPS variants, thereby reducing systemic inflammation (110).By dampening microbiome-derived endotoxemia, such interventions can help recalibrate the systemic immune setpoint, thereby delaying the exhaustion of tumor-infiltrating CD8^+^ T cells and potentially extending the durability of ICB therapies.

### 4.3 LPS-driven immune evasion in the TME

Within the TME, LPS exerts context-dependent immunomodulatory effects, primarily mediated by TLR4 signaling in epithelial and myeloid compartments (111–113). Chronic endotoxin exposure typically fosters a highly immunosuppressive niche. In the hepatic TME, persistent LPS signaling can drive the functional exhaustion of CD8^+^ T cells, marked by upregulation of inhibitory receptors such as PD-1 and TIM-3.signaling Concurrently, this chronic inflammatory state triggers anti-apoptotic signaling and genotoxicity in hepatocytes, thereby promoting hepatocellular carcinoma (HCC) progression (114, 115).Similar immunosuppressive networks are mediated by LPS across diverse gastrointestinal malignancies. In cholangiocarcinoma, LPS from Gram-negative bacteria activates TLR4-dependent CXCL1 secretion, thereby recruiting CXCR2^+^ PMN-MDSCs that suppress local antitumor immunity (6) (**Figure 5**). In right-sided colon adenomas, LPS-induced S100A11–RAGE signaling recruits MDSCs and markedly suppresses CD8^+^ T cell effector functions, contributing to a microenvironment associated with poor clinical prognosis (7).At the molecular level, LPS can upregulate the long non-coding RNA LINC00152 through histone lactylation modifications in its promoter region, while reducing binding of the repressor YY1, thereby promoting colorectal cancer cell migration and invasion (116).

### 4.4 Microbiota-LPS Crosstalk and Immunotherapeutic Potential

The structural diversity of LPS produced by different commensal and pathogenic bacteria underpins the highly divergent functional outcomes observed in tumor immunity (117). For instance, LPS derived from pathogenic bacteria such as *Fusobacterium nucleatum* can upregulate BIRC3 expression through the TLR4/NF-κB axis, conferring chemoresistance to 5-fluorouracil in colorectal cancer (118). In addition, specific LPS variants from *Prevotella* and *Porphyromonas* species can promote an immunosuppressive microenvironment by suppressing cytokine secretion from peripheral blood mononuclear cells, and have been associated with the distinct clinical trajectories of the CMS1 and CMS4 molecular subtypes of colorectal cancer (119–121). However, not all LPS variants exert deleterious effects. LPS variants from certain commensal bacteria (e.g., those with specific hexosylation modifications) can markedly activate TLR4 and, in preclinical models, synergize with PD−1 inhibitors to enhance the infiltration of IFN−γ^+^CD8^+^ T cells and induce Bax−mediated apoptosis, thereby inhibiting tumor growth and sensitizing ICB therapy (122, 123). Therefore, simplistically categorizing LPS as either a “pro-tumor endotoxin” or an “immunoadjuvant” is reductive, as its function is highly dependent on its structural origin and microenvironmental context. Leveraging this microbial–endotoxin diversity, targeted pharmacological interventions can selectively reshape the LPS–microbiota axis. For example, glycyrrhizin derivatives have been shown to suppress pathogenic Escherichia coli while selectively expanding beneficial commensals such as *Akkermansia*, reversing M2 macrophage polarization, enhancing NK and T cell infiltration, and limiting tumor burden (123). These findings support the translational feasibility of manipulating LPS signaling in precision immunotherapy. Furthermore, chemically modified LPS derivatives (e.g., MPL) have already been clinically applied as vaccine adjuvants, and their potential in tumor immunotherapy remains to be systematically explored (104).

## 5 Cancer therapies reshape the gut microbiota: evidence for bidirectional regulation

The relationship between the gut microbiota and cancer therapy is bidirectional. Previous studies have largely focused on the modulatory effects of the microbiota on responses to chemotherapy, immune checkpoint inhibitors, and radiotherapy. However, emerging evidence indicates that these therapeutic modalities can also reshape the composition and metabolic functions of the gut microbiota, thereby potentially establishing a new microbial baseline for subsequent treatment. Understanding this bidirectional regulatory relationship has implications for the dynamic interpretation of microbiota-related biomarkers and the design of microbiota-guided sequential intervention strategies.

### 5.1 Immune checkpoint inhibitors

As a therapeutic approach that relies on activating the host’s own immune defense mechanisms to combat malignancies, immune checkpoint blockade has substantially changed traditional cancer treatment paradigms and has been associated with disease control in patients with advanced cancer (124). Nevertheless, inter-individual variability in treatment efficacy has been observed in clinical practice, and this variability can be partially attributed to differences in gut microbiota composition. A body of evidence has confirmed that specific microbial signatures are associated with the efficacy of anti-PD-1/PD-L1 and anti-CTLA-4 therapies as well as the occurrence of immune-related adverse events (125).

Concurrently, ICB therapy itself can exert selective pressure on the gut microbiota by modulating the host immune status. At the mechanistic level, anti-CTLA-4 antibodies have been reported to induce the differentiation of pro-inflammatory Th17 cells in the intestinal mucosa, thereby influencing the microenvironmental conditions for local microbial colonization (125). In a longitudinal analysis of fecal microbiota in a small cohort of melanoma patients receiving anti−PD−1 therapy, Frankel et al. observed that the relative abundance of genera such as *Faecalibacterium* changed after treatment, and these changes were associated with treatment response status. In addition, ICB−induced alterations in the intestinal mucosal cytokine profile (such as changes in IFN−γ and IL−10 levels) may indirectly influence the colonization environment of commensal bacteria (126). In preclinical models, Mager et al. demonstrated that *Bifidobacterium* can enhance anti-PD-1 efficacy by cross-activating dendritic cells through expression of specific antigens; conversely, ICB therapy may also modulate the intestinal immune environment to alter the colonization density of Bifidobacterium and other strains (127). Beyond changes in bacterial abundance, ICB therapy has been shown to selectively modulate specific microbial functional pathways. Multi-omics analyses of ICB-treated patients have revealed that anti-PD-1/PD-L1 therapy is associated with distinct microbial metabolic pathway signatures, with the MEP pathway (involved in Vδ2 T cell-mediated antitumor responses) and riboflavin synthesis pathway (associated with MAIT cell-mediated immunosuppression) showing differential enrichment between responders and non-responders (128, 129). Furthermore, integrative microbiome and metabolome profiling has identified metabolite correlates of ICB responses, such as phenylacetylglutamine (PAGln), which was negatively associated with anti-PD-1 efficacy in a clinical cohort and has been shown to impair antitumor immune responses in subsequent *in vivo* experiments (94). Beyond changes in metabolites produced by the microbiota, ICB−induced shifts in the intestinal metabolome may also modulate the balance between Tregs and effector T cells through FXR and TGR5 signaling, as bile acid profiles are known to influence the differentiation and functional states of these immune populations (130).

These shifts in microbial community structure may lead to the reorganization of metabolite profiles, including SCFAs and BAs, which may in turn feedback to influence immune responses to subsequent treatment. It should be noted, however, that evidence for a causal relationship between microbial dynamics during ICB therapy and clinical outcomes remains limited; most conclusions are drawn from correlative studies or preclinical models, and further prospective cohort and mechanistic studies are warranted.

### 5.2 Chemotherapy-induced microbial remodeling with immunomodulatory consequences

Chemotherapy can reshape the gut microbiota and, in some cases, produce immunomodulatory effects that extend beyond direct cytotoxicity. A recent study in colorectal cancer models demonstrated that chemotherapy−induced intestinal mucositis alters the luminal nutrient environment, promoting the expansion of tryptophan−metabolizing bacteria and the production of the microbial metabolite indole−3−propionic acid (IPA). IPA has been reported to reprogram bone marrow myelopoiesis, modulating differentiation toward macrophages and reducing the generation of immunosuppressive Ly6C^high^ CCR2^+^ monocytes, potentially enhancing the antitumor function of CD4^+^ T cells. Consistently, elevated circulating IPA levels after chemotherapy were associated with lower monocyte abundance and improved survival in colorectal cancer patients (131). This example illustrates that chemotherapy-driven microbial remodeling can yield immunological benefits through metabolite-mediated mechanisms, which may also influence the efficacy of subsequent immunotherapy.

### 5.3 Radiotherapy−induced gut dysbiosis: potential immunomodulatory implications

Radiotherapy can disrupt the gut microbiota through damage and immune-mediated mechanisms (132).

Clinical and preclinical studies have documented dose- and regimen-dependent shifts in microbial composition (133–135).However, evidence that these radiotherapy−induced microbial changes modulate antitumor immune responses or immunotherapy outcomes remains scarce. Some reports note associations with systemic inflammatory markers (e.g., IL-6) (136).suggesting a potential link to immune regulation, but causal relationships in the tumor−immunity context have yet to be established. Given the expanding use of combined radiotherapy and immunotherapy, future studies are needed to determine whether radiotherapy−induced microbial alterations can be therapeutically exploited to enhance immune checkpoint blockade or adoptive cell therapies.

## 6 Conclusions and future perspectives

The metabolites systematically reviewed herein, along with the cell wall component LPS, represent a range of microbiota-derived molecules that exert context-dependent dual effects within the TME—either modulating antitumor immunity or contributing to immune evasion. This does not reflect a flaw in the research, but rather the inherent nature of these molecules as “context-dependent messengers”: their functions are not intrinsic attributes, but rather dynamic composite biomarkers co-defined by the host microenvironment (immune cell composition, cytokine profiles), local concentration, receptor expression profiles, and microbial context (137–139). To move beyond descriptive summary, we constructed a cross-comparison framework (**Supplementary Table 1**) annotating each metabolite’s pro-tumor and antitumor effects by tumor type, anatomical site, receptor context, and concentration. This analysis revealed four predictive patterns. First, a gastrointestinal vs. extra-intestinal axis appears to govern TMAO: it drives NLRP3-dependent immunosuppression in colorectal cancer, yet has been reported to activate PERK-dependent immunogenic cell death or pyroptosis in pancreatic, breast, and melanoma models, enhancing ICB. Bile acids show a parallel divergence—hydrophobic DCA/LCA promote immunosuppression in liver and gastrointestinal tumors, whereas hydrophilic UDCA has been reported to exert antitumor effects in prostate cancer. Second, cell death pathway divergence appears to determine net outcome: TMAO engages NLRP3 in colorectal cancer (pro-tumor) but PERK–GSDME pyroptosis in triple-negative breast cancer (antitumor), with PERK itself producing opposite immunological outcomes in tumors versus the cardiovascular system. Third, a therapeutic modality axis appears to govern butyrate: it sensitizes chemotherapy and ICB via HDAC inhibition, yet has been shown to suppress STING–Type I interferon signaling to antagonize radiotherapy. Fourth, the structural origin of LPS—pathogen-derived vs. commensal-derived—may determine whether it drives T cell exhaustion and chemoresistance or enhances ICB, mirroring the species-dependent divergence of bile acids. For several emerging metabolites, only unidirectional effects have been reported to date; systematic testing under contrasting conditions is needed to establish the generality of this framework. These patterns identify knowledge gaps—particularly quantitative concentration–response data across tumor types and paired receptor expression profiling—that need to be addressed for precision targeting of the metabolite–immunity axis.

Although the role of the gut microbiota in tumor immunotherapy has been widely recognized, its clinical translation still faces challenges and contradictions. First, whether specific microbial features (e.g., *Akkermansia*) can universally predict immunotherapy responses across cancer types or are tumor-specific remains unclear. Second, the effects of SCFAs are contradictory—they can both enhance CD8^+^ T cell function to improve ICB efficacy and impair antitumor immunity by inhibiting T cell migration into tumor tissues. Third, most studies have focused on baseline microbiome composition, whereas whether dynamic changes in the microbiota during ICB treatment predict patient outcomes better than static measurements remains insufficiently investigated. These insights have implications for translational strategies: pursuing “one-size-fits-all” interventions (such as simple butyrate supplementation, TMAO inhibition, or comprehensive LPS signaling blockade) may be counterproductive, and selective precision modulation based on patient multi-omics stratification (metabolic profiles, immune profiles, microbial profiles) is required. By integrating microbial features with genomic and immune profiling, composite models may enable accurate prediction of ICB responses and stratification according to the risk of immune-related adverse events (140). Concurrently, resolving the receptor structures of these molecules, developing tissue-specific delivery systems, and establishing integrative models to predict net effects represent advances toward clinical translation in this field (141).

At the translational application level, intervention strategies targeting the gut microbiota are advancing from preclinical research toward early-stage clinical trials. Representative registered clinical trials investigating microbiota- and metabolite-based cancer interventions are summarized in **Table 2**. FMT has shown potential to enhance immunotherapy responses in multiple studies: A recent systematic review and meta-analysis of 12 mostly phase I, single-arm trials (total n=180) reported a pooled ORR of 33.7% (95% CI 18.1–53.9%) for immunotherapy combined with FMT, with the first-line subgroup reaching an ORR of 59% and a DCR of 80.4%. All included studies were non-randomized, and the confidence interval suggests inter-study heterogeneity; these early-phase results, while promising, should therefore be interpreted with caution pending validation in larger randomized cohorts (142). The MIMic Phase I trial—a small (n=20), single-arm study in advanced melanoma—demonstrated that healthy donor FMT combined with first-line anti-PD-1 therapy was safe and feasible, with an ORR of 65% and median PFS and OS of 29.6 and 52.8 months, respectively. These outcomes are encouraging but must be considered hypothesis-generating, given the small sample size and lack of a control arm, and require confirmation in randomized trials (143). Overall, both lines of evidence remain early-phase, and conclusions will require confirmation in larger randomized studies. Postbiotics, as bioactive metabolites produced by probiotic fermentation, have entered the clinical trial stage (NCT07600645, ChiCTR2500110570). In the field of engineered bacteria, a first-in-human, phase I dose−escalation trial of intratumoral *Clostridium novyi−NT* spores in 24 patients with treatment−refractory advanced solid tumors demonstrated tumor regression at injected sites in 41% of evaluable patients, with evidence of intratumoral spore germination and tumor lysis in 42% of participants. However, dose-limiting toxicities, including grade 3 sepsis and gas gangrene, were observed, highlighting the narrow therapeutic window and the need for further safety optimization. Bacillus Calmette–Guérin (BCG) remains the only bacterial preparation approved by the FDA for cancer therapy, but is limited to intravesical instillation for non−muscle-invasive bladder cancer. Therefore, the safety and efficacy of bacterial−based cancer therapies require further clinical validation (141).

**Table 2:** Summary of clinical trials investigating the role of gut microbiota and metabolites in cancer therapy.

Associated tumor type	Trial number	Specific interventions/assessment methods	Primary endpoints/preliminary outcomes
Glioma	ChiCTR2300067789	Fecal and tumor tissue sampling for multi-omics sequencing	Identification of intratumoral microbiota and correlation of fecal microbiota profiles with glioma progression
Colorectal Cancer (Adenoma-Carcinoma)	ChiCTR2300068010	Multi-segment colonic microbiota sampling and metagenomic profiling	Characterization of microbial diversity shifts driving the adenoma-carcinoma evolutionary sequence
Biliary Tract Tumor	ChiCTR2300068817	Serum and fecal bile acid targeted metabolomics	Identification of specific bile acid signatures correlated with tumor diagnosis and disease progression
Hepatocellular Carcinoma	ChiCTR2200061688	Administration of specific Traditional Chinese Medicine (TCM) formulations	antitumor efficacy evaluation and dynamic modulation of the Bacteroidetes-FXR bile acid axis
Colorectal Cancer	ChiCTR2100053715	Preoperative oral supplementation of branched-chain amino acids (BCAAs)	Assessment of postoperative insulin resistance attenuation and systemic metabolic recovery
Metastatic Colorectal Cancer	ChiCTR2500103189	Oral nutritional supplementation with β-hydroxy-β-methylbutyrate (HMB)	Incidence reduction and clinical improvement of sarcopenia in patients with primary advanced cancer
Colorectal Cancer	ChiCTR1900028063	Fecal/tissue sampling from patients receiving anti-PD-1 or Bevacizumab therapy	Correlation of specific bacteria (e.g., Fusobacterium nucleatum) with the objective response rate (ORR) of immunotherapy

ChiCTR, Chinese Clinical Trial Registry; BCAAs, branched-chain amino acids; FXR, farnesoid X receptor; PD-1, programmed death-1; HCC, hepatocellular carcinoma; HMB, β-hydroxy-β-methylbutyrate; ORR, objective response rate; PMN-MDSCs, polymorphonuclear myeloid-derived suppressor cells.

Most microbiota-based intervention strategies remain at an early stage with limited sample sizes, and their efficacy and long-term safety require confirmation through larger-scale randomized controlled trials. Implementing rigorous standards is essential to ensure reproducibility: clinical trials must control for confounding factors and standardize analytical resources to isolate causal drivers from noise, which is a fundamental prerequisite for regulatory approval (144).

Overall, treating the patient and their microbial ecosystem as a unified biological entity is emerging as a new perspective in tumor immunotherapy research. Precision strategies targeting microbial metabolites and the microbiome are still under exploration, and their clinical value awaits confirmation through additional studies (145). Through the establishment of standardized methodologies, mechanistic investigation, advancement of early-phase clinical exploration, and integration of multidimensional patient data can this field be translated from descriptive associations into clinical application.
